# Annotations

**Published:** 1898-07-16

**Authors:** 


### Annotations.

### The Inspection of Meat.

The tables given in the annual report of the Director of the Leipzig Abattoir for 1897, as published in *Public Health*, give much interesting information regarding the results of the system of inspection adopted there. It will be remembered that at Leipzig it is not the custom to destroy the whole of the carcase of every tuberculous animal. A careful inspection is made, and according to the degree to which the disease is found to have extended the meat is either allowed to be sold without any restriction, or only the fat is melted out and sold, or the meat is sold for food after being sterilised or cooked, or is sold in the raw state as of second quality, or finally is totally condemned. This being understood, it is interesting to note that of 27,191 cattle slaughtered, 9,899, or 36·4 per cent., were tuberculous. In the great mass of these cases, however, the disease was localised, in 7,018 of them occurring in one organ only. Thus it happened that the flesh of 92·19 per cent. of the cattle in whom tuberculosis was discovered was sold for food without any restriction, and that of 5·73 per cent. was sold after sterilisation or as second class meat, and that the flesh of only 2·08 per cent. of the cattle found to be tuberculous was totally condemned. In pigs the proportions were rather different. A far smaller proportion of pigs than of cattle were affected with tuberculosis, but those pigs that were affected showed a much greater tendency to generalisation, so that the flesh of only 59·84 per cent. of the tuberculous pigs was sold without restriction. On the other hand very few pigs were totally condemned, which is rather curious in view of the comparative frequency with which the disease extended to the glands of the muscular system. The moral we draw from these figures is that a careful inspection pays for itself, by saving, as good useful food, a very large quantity of meat which under a more rule-of-thumb arrangement might be thrown away.

### The Test of a Good Citizen.

The discussion in the House of Lords on the Aliens Bill raised the very interesting question whether a man is best off with money or with brains. We should not have thought that the problem was one worthy of a minute's disputation. Yet when it was proposed to remove the words which make the Act apply to "a person without means of support," on the ground that as these words stand they tend, as Lord Cowper puts it, to admit a man who might physically be of little value, while they exclude a man with a strong right arm and a clear head if he has no money with him, no one stood up to say that a "strong right arm and a clear head" are, in fact, a means of support. We cannot but feel with Lord Kelvin that it is a serious matter that an Act should be passed in such a form that able-bodied young men arriving without money might be refused admission to this country. Lord Herries distinctly made a point when he drew attention to the fact that technical training had been largely fostered on the Continent, and that many workmen who came here with all the knowledge which they had acquired in Continental schools really contributed to the wealth of the country, even although they landed without a penny in their pockets. But he might have gone further, for there is a form of wealth that cannot be expressed in terms of gold, and it is of far more importance to us as Englishmen that aliens who come over to mingle with our race should possess firm brains, strong muscles, and good health, than that they should have a little money in their pockets. What we have to recognise is that the English race is a mixed breed, and that, if we are to look to the good of England rather than the immediate prosperity of the present possessors of the labour market, any regulation which regulates the admission of aliens on a purely money basis is likely to lead to evil rather than good. Bodily health and that degree of mental health which is shown by absence of criminality are of much more importance than the possession of a little money, and we should have welcomed some assurance that a man with a "strong arm and a clear head" would be admitted even if he came empty-handed. It is the mentally and the bodily unfit that we should exclude.

### Water Companies and Sanitary Authorities.

The education of the people and of their representatives in regard to matters of water supply is a slow and weary process. The lesson of Maidstone seems already to be forgotten, and the exigencies of parliamentary life and party government have actually made it necessary for Mr. Chaplin to withdraw his proposals giving power to a sanitary authority, whose district is supplied by a water company, to take samples of water from any point within the company's works, and enabling any such company to enter upon lands from which their water is derived, and to take samples from points where it might seem likely to become polluted. When such things happen it is impossible to believe that either voters or members of Parliament realise the dangers of impure water. When people see published in the papers what appear as official reports as to the condition of the water supplies, they are all too apt to imagine that our officials enter as of right, and take their samples where they like; but this is far from being the case. At the present time even in London the sanitary authority has not only no control over the arrangements by which drinking water is manufactured from the raw material—the washings of the Thames Valley—as it flows down the river-bed, but have not even the power to enter and see that such processes as the companies choose to employ for the purpose are being properly carried out. This, no doubt, is a surprising condition of affairs, but then the relations between politics and sanitation are generally of a somewhat surprising character. We do not say a word here against the principle of public water supplies being undertaken by trading companies—that is quite another question; but we do say that the sanitary authorities ought to have the power of ascertaining whether the drinking water supplied is being properly purified or not; and even that little favour is more than is granted at present. Everything nowadays is done in the name of sanitation; but when, in the name of sanitation, we ask for some clean water, we are told to open our mouths and shut our eyes, like good little children, and take what is given us! Do we, then, protest against being treated in this ignominious fashion? Not a bit of it! We pay our taxes manfully, and trust that the Government, or the corporation, or the vestry, or someone in an office somewhere or other will look after us. Such faith is, indeed, childlike.

